# Cellular and Cytokine Responses to *Salmonella enterica* Serotype Typhi Proteins in Patients with Typhoid Fever in Bangladesh

**DOI:** 10.4269/ajtmh.13-0261

**Published:** 2014-06-04

**Authors:** Saruar Bhuiyan, Abu Sayeed, Farhana Khanam, Daniel T. Leung, Taufiqur Rahman Bhuiyan, Alaullah Sheikh, Umme Salma, Regina C. LaRocque, Jason B. Harris, Marcin Pacek, Stephen B. Calderwood, Joshua LaBaer, Edward T. Ryan, Firdausi Qadri, Richelle C. Charles

**Affiliations:** Division of Vaccine Sciences, International Centre For Diarrhoeal Disease Research, Bangladesh, (icddr,b), Dhaka, Bangladesh; Division of Infectious Diseases, Massachusetts General Hospital, Boston, Massachusetts; Department of Medicine, Harvard Medical School, Boston, Massachusetts; Department of Microbiology and Immunobiology, Harvard Medical School, Boston, Massachusetts; Virginia G. Piper Center for Personalized Diagnostics, Biodesign Institute, Arizona State University, Tempe, Arizona; Department of Immunology and Infectious Diseases, Harvard School of Public Health, Boston, Massachusetts

## Abstract

We assessed interferon-gamma (IFN-γ) responses via enzyme-linked immunosorbent spot (ELISPOT) to a number of *S.* Typhi antigens in samples from humans with *S.* Typhi bacteremia and typhoid fever in Bangladesh. Compared with responses in healthy endemic zone controls, there were significantly increased IFN-γ responses at the time of clinical presentation (acute phase) and at convalescence 14–28 days later. The majority (80–90%) of IFN-γ expressing T cells were CD4+. We observed a significant increase in interleukin-17 (IL-17) positive CD4 + T cells at convalescent versus acute stage of infection using an intracellular cytokine staining assay. We also found that stimulated peripheral blood mononuclear cells (PBMCs) produced significantly increased levels of a number of cytokines at the convalescent versus acute phase of infection, including IFN-γ, MIP-1β, sCD40L, TNF-β, IL-13, and IL-9. These results suggest that *S.* Typhi antigens induce a predominantly Th1 response, but that elevations in other cytokines may be modulatory.

## Introduction

Typhoid fever is a systemic illness caused by infection with *Salmonella enterica* serotype Typhi, a Gram-negative intracellular bacterium. Worldwide, typhoid fever affects an estimated 21 million people each year, causing over 200,000 deaths, with the highest incidence in South Central and Southeast Asia.^1^ *Salmonella* Typhi is a human-restricted pathogen, a reality that has complicated advancing our understanding of host–pathogen interactions. Specifically, data on human immune responses during wild-type typhoid fever in humans are sparse, especially data on cellular responses that may be critical in controlling and clearing *S.* Typhi infection.

Volunteer challenge studies of wild-type *S.* Typhi infection performed in the 1960s and 1970s suggested a key role for cellular immune responses; however, these studies were performed before the availability of modern immunologic techniques.^2^ Our current understanding of cellular responses during *S.* Typhi infection are extrapolated from murine models of *S.* Typhimurium infection (that cause a systemic typhoidal-like illness in mice), and from human studies with attenuated *S.* Typhi live vaccine strains.^3^ From murine studies with *S.* Typhimurium, it has been shown that interferon-gamma (IFN-γ) produced by Th1 cells plays an important role in bacterial killing, and depletion of Th1-associated cytokines (i.e., IFN-γ, interleukin-12 [IL-12], or tumor necrosis factor α [TNF-α]) reduces the protective immune response conferred by attenuated live *Salmonella* vaccines.^4^ In healthy North American volunteers, oral ingestion of live attenuated *S.* Typhi strains has been shown to elicit CD4 and CD8 T cell responses, with induction of Th1 cytokine responses including IFN-γ and TNF-α to *S.* Typhi antigens.^5^,^6^

We have previously shown in patients with wild-type typhoid fever in Bangladesh that lymphocytes stimulated with *S.* Typhi antigens produce strong IFN-γ CD4 responses.^7^ Here, we report a further assessment of the cellular and cytokine responses in patients with typhoid fever after stimulation with a number of *S.* Typhi antigens, including those identified by a high-throughput screen to identify proteins that generate an immune response.^8^

## Materials and Methods

### Study participants.

We enrolled individuals (2–17 years of age) who presented to either the Kamalapur field station or Dhaka hospital of the International Centre for Diarrheal Disease Research, Bangladesh (icddr,b) in Dhaka. The Kamalapur field site is a clinic located in an urban community mostly consisting of urban slums. We collected venous blood from patients presenting with 3–5 days of fever for whom there was clinical suspicion of enteric fever by study clinic staff. Clinical features of study participants are listed in Table 1 ; we collected venous blood in tryptic soy broth (TSB) blood culture bottles and incubated these in a BacT/Alert automated system (bioMerieux, New Delhi, India). Positive cultures were confirmed by *S.* Typhi antisera agglutination and standard biochemical methods.^9^ We confirmed *S.* Typhi bacteremia in 10 patients, and obtained additional samples from those patients at acute (2–3 days later) and convalescent (14–28 days later) stages of illness. Patients were treated at the discretion of the attending physician, usually receiving oral cefixime or parenteral ceftriaxone. All patients recovered. We also obtained blood samples from 10 healthy Bangladeshi volunteers as endemic zone healthy controls. This study was approved by the Ethical Research Committee of the icddr,b and the Institutional Review Board of the Massachusetts General Hospital. Informed consent was obtained from all adult participants and from parents or legal guardians of minors.

### Isolation of peripheral blood mononuclear cells (PBMCs).

We separated PBMCs from diluted heparinized blood by Ficoll-Isopaque (Pharmacia, Piscataway, NJ) density gradient centrifugation. We washed isolated PBMCs in phosphate buffered saline (PBS, pH 7.2) and resuspended them in RPMI-1640 media (Gibco, Carlsbad, CA) supplemented with 10% heat-inactivated fetal bovine serum (FBS) (Hyclone-Thermo Scientific, Waltham, MA), 100 U/mL penicillin, 100 μg/mL streptomycin, 100 mM pyruvate, and 200 mM L-glutamine (Gibco).

### Proteins used for measuring immune responses.

For assessing IFN-γ responses by enzyme-linked immunosorbent spot (ELISPOT), we used the following *S.* Typhi proteins purified in 96-well plates using a Biomek FX robotic liquid handler as previously described^7^: flagellar protein (FliC), chaperone protein EcpD (STY0206, StaB), fimbria-like protein FimF precursor (STY0595), conserved hypothetical protein (STY0909), phage shock protein E precursor (STY1375, PspE), putative secreted hydrolase (STY1522), putative fimbrial subunit protein (STY2381, StcA), fimbrial subunits (STY3089 and STY3090, SteEF), and autoinducer 2 import ATP-binding protein (STY3796, LsrA) (Table 2 ). We also extended our analysis of SteE (STY3089) and CsgD (STY1179) using recombinant antigens prepared as His-fusion proteins expressed from pDEST17 Gateway-based cloning vector (Invitrogen Life Technologies, Carlsbad, CA) and recovered by affinity chromatography.^10^ We focused our analysis on these 10 proteins because they (or the operons in which they are contained) had been previously identified by an immunoscreen (*in vivo*-induced antigen technology) using sera from patients with acute typhoid infection or were related (i.e., fimbrial subunit).^8^ We also prepared membrane protein (MP) and lipopolysaccharide (LPS) produced from *S.* Typhi Ty21a as target antigens as previously described.^7^,^11^–^13^ We used phytohemagglutinin (PHA) as a positive control, and keyhole limpet hemocyanin (KLH) as a negative control.

### IFN-γ ELISPOT assay.

We assessed IFN-γ expression in acute and convalescent phases of infection using an ELISPOT assay (MabTec Inc., Cincinnati, OH) in eight patients with *S.* Typhi bacteremia, according to manufacturer's instructions and as described previously.^7^ Briefly, we coated ELISPOT plates (Multiscreen HTS, Millipore, Billerica, MA) with 100 μL of 15 μg/mL human monoclonal anti-IFN-γ antibody (1-DIK) overnight at 4°C. We washed the plates and blocked with 10% FBS for 2 hours at room temperature. We added fresh PBMCs to the wells at a concentration of 2 × 10^5^ per well for each antigen. We added *S.* Typhi antigen or control at a concentration of 140 ng/well of total preparation for each purified antigen, making the final concentration 0.7 μg/mL. We also added MP to ELISPOT plates at a concentration of 10 μg/mL in 200 μL culture, and PHA (Murex Diagnostics Ltd., Temple Hill, UK), at a final concentration of 2.5 μg/mL, as a positive control. We used KLH as a negative control. We incubated the plates for 20 hours at 37°C in 5% CO_2_, washed them, and added anti-IFN-γ antibody (7-B6-1-biotin; 1:500 dilution). We incubated the plates for 2 hours at room temperature, washed them, added streptavidin-HRP (1:500 dilution), and incubated the plates again for 1 hour at room temperature. We developed the plates with aminoethylcarbazole with H_2_O_2_, and then washed and dried them. We counted the spots under a stereomicroscope, and expressed results as the number of spots/10^6^ PBMCs, subtracting the number of spots in media only wells from spots in antigen-specific wells. We compared differences between groups using the Wilcoxon signed-rank test.

### Intracellular cytokine staining.

We characterized cytokine production in six patients by intracellular cytokine staining (ICS), as described previously.^7^ Briefly, we resuspended PBMCs at a concentration of 10^6^ cells/mL in RPMI medium (Gibco) supplemented with 10% fetal calf serum (FCS, Gibco). We cultured these PBMCs in U-bottom culture plates in the presence of *S.* Typhi Ty21a MP, STY3089, STY1179, *S.* TyphiTy21a LPS, phorbol 12-myristate 13-acetate (PMA), or without stimulation. We added co-stimulatory molecules anti-CD28 (1.0 mg/mL; clone 28.2; BD Pharmingen, San Jose, CA) and anti-CD49d (1.0 mg/mL; clone 9F10; BD Pharmingen) to the culture and incubated at 37°C with 5% CO_2_. After 2 hours of incubation, we added brefeldin A (Sigma, St. Louis, MO) at a concentration of 10 μg/mL and incubated for another 4 hours. We washed the cells with PBS and 2% FCS. We then stained cells for 30 min at 4°C with cell surface antibodies anti-CD3-PE-Cy7, anti-CD4-Amcyan, anti-CD8-PE Texas red, anti-CD14-APC-Cy7, and anti-CD56-PerCP-Cy5. We lysed red cells using a FACS lysing solution (BD Bioscience, San Jose, CA) for 10 minutes, and then permeabilized cells using FACS permeabilizing solution (BD Bioscience) for 10 minutes in a dark room. We washed the permeabilized cells and incubated them with anti-IL-13-PE, IL-10-APC, IFN-γ-V450, and IL-17a-Alexa Fluor488 (BD Bioscience) for 45 min. We then washed and fixed the cells in cell fix buffer (BD Bioscience), and kept cells at 4°C until flow cytometry was performed. We focused our analysis on these 4 pro-inflammatory regulatory cytokines as surrogate markers of Th1 (IFN-γ), Th2 (IL-13), Treg (IL-10), and Th17 (IL-17) responses. We performed flow cytometry with a FACSAriaIII (BD Bioscience). We identified the lymphocyte population on forward versus side scatter plot, and then we gated for CD3+ sub-populations. We used Flow-Jo software for flow cytometry data analysis (Treestar, Ashland, OR). We subtracted unstimulated responses from the antigen-specific responses and performed statistical comparison using the Student's *t-*test.

### Cytokine assay by Luminex.

We cultured PBMCs from three bacteremic patients with MP antigen and collected culture supernatant after 48 hours of incubation at 37°C in 5% CO_2_. We measured concentrations of cytokines using a Luminex human cytokine/chemokine assay kit (Millipore) according to manufacturer's instructions^14^; briefly, we filtered culture supernatant through a nitrocellulose membrane to remove debris. We prepared standards in Assay Buffer in 10-fold dilutions, and added 25 μL of each standard to a final volume with media of 50 μL. In sample wells, we added 25 μL of assay buffer and 25 μL of culture supernatant to make a final volume of 50 μL. We incubated the plates at room temperature for 1 hour with shaking, and gently removed fluid by vacuum and washed the wells twice with wash buffer. We then added 25 μL of detection antibody to each well, covered the plate with aluminum foil, and incubated it for 1 hour at room temperature. After incubation, we added 25 μL streptavidin-phycoerythrin to each well, and incubated the plates for another 30 minutes with shaking. After washing, we added 150 μL of FACS fluid (BD Biosciences) to each well and analyzed results in Luminex 100 IS (Bio-Rad, Hercules, CA). We subtracted unstimulated cytokine values (pg/mL) from the stimulated value to calculate net stimulation, log transformed the data, and compared means using a paired *t*-test. We calculated relative change by dividing mean net stimulation at convalescent stage by mean net stimulation at acute stage.

## Results

### IFN-γ responses by ELISPOT.

**Figure 1.**
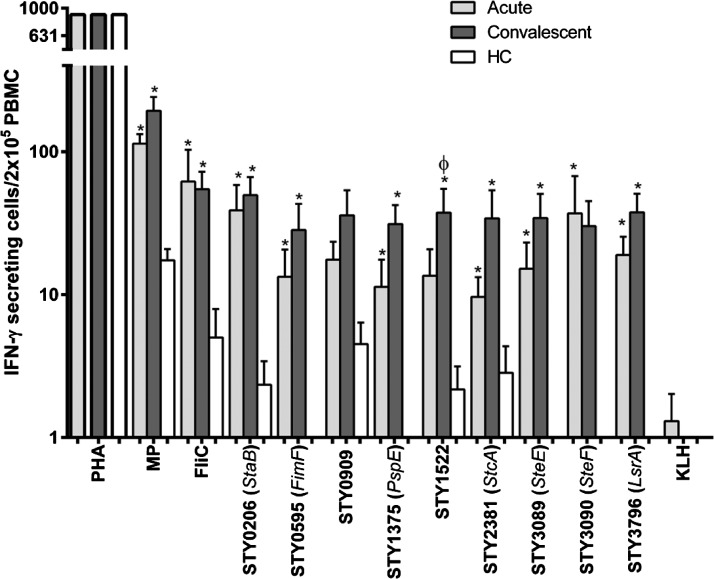
Interferon-gamma (IFN-γ) enzyme-linked immunosorbent spot (ELISPOT) responses to *S.* Typhi antigens in peripheral blood mononuclear cells (PBMCs) from patients with typhoid fever at acute and convalescent phases of illness. KLH = keyhole limpet hemocyanin; PHA = phytohaemagglutinin; HC = healthy controls; MP = membrane preparation. * indicates statistically significant increase (*P* < 0.05) at acute and convalescent phase of illness in typhoid fever patients compared with healthy controls. Φ indicates statistically significant increase (*P* < 0.05) at acute compared with convalescent phase of illness in typhoid fever patients.

Using the ELISPOT method, we found significantly increased IFN-γ responses at both acute and convalescent stages of infection upon stimulation with all *S.* Typhi proteins except STY0909, compared with endemic-zone healthy controls (Figure 1). We did note a significant increase in IFN-γ response in convalescent compared with acute samples for STY1522 (a putative secreted hydrolase, *P* = 0.03); and a trend was seen for Phage shock protein E (STY 1375, *P* = 0.06) and a putative fimbrial subunit, StcA (STY2381, *P* = 0.08). Minimal responses were detected to our negative control (KLH), and responses to the positive control (PHA) did not differ significantly between patients or healthy controls.

### CD4 and CD8 responses.

**Figure 2.**
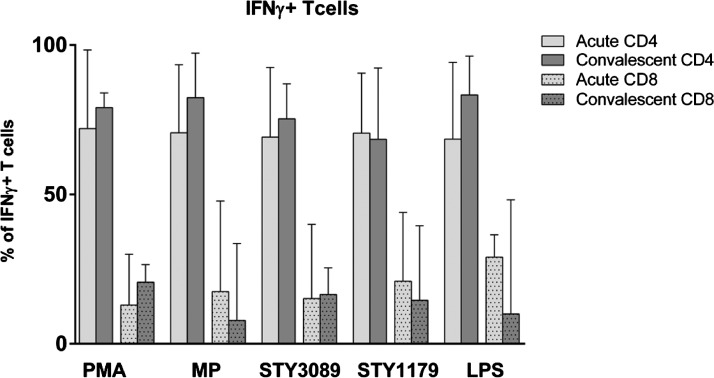
Characterization of interferon-gamma (IFN-γ) producing T cells during stimulation with typhoid antigens, expressed as a percentage of total CD4 and CD8 cells at acute and convalescent phases of illness. PMA = phorbol myristate acetate; MP = membrane preparation; LPS = lipopolysaccharide; IFN-γ, interferon-gamma.

**Figure 3.**
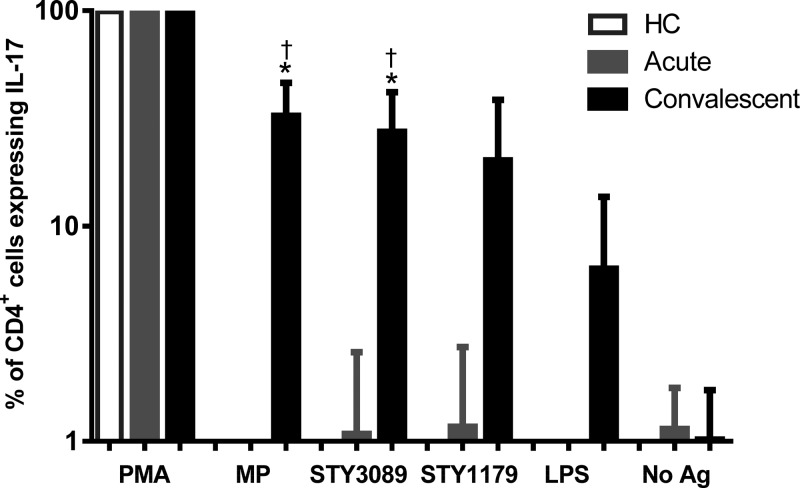
Interleukin-17 (IL-17) responses in CD4 cells assessed by intracellular cytokine staining assay following stimulation with membrane preparation (MP), fimbrial protein (STY3089), CsgD (STY1179), LPS, phorbol, 12-myristate 13-acetate (PMA), or no antigen (No Ag). * Indicates statistically significant increase (*P* < 0.05) from acute to convalescent phase of illness in patients with typhoid fever and † indicates statistically significant increase at convalescent phase compared with healthy controls.

Using intracellular staining and flow cytometry, we found that the majority (60–80%) of cells producing IFN-γ in response to all antigens tested at both the acute stage and convalescence were CD4+ T cells, with a smaller number (10–20%) of CD8+ cells (Figure 2

). The IL-17 responses in CD4+ cells to *S.* Typhi antigens MP and STY3089 were also detectable at convalescence, but not during the acute stage of illness (Figure 3

). We did not see any intracellular expression of IL-10 or IL-13 in CD4+ or CD8+ cells.

### Cytokine responses in culture supernatants.

To further characterize the cytokine responses during the acute versus convalescent stages of infection, we used the human cytokine/chemokine premixed multiplex assay (Millipore) to measure the concentration of 39 cytokines in culture supernatants of PBMCs stimulated with *S.* Typhi MP antigen for 48 hours (Supplemental Table 1). We found significant increases for the following cytokines expressed from stimulated PBMCs in convalescent versus acute stages of illness: IFN-γ, MIP-1β, IL-13, sCD40L, Fractalkin, IL-9, TNF-β, Eotaxin, and IL-15 (Table 3 ).

## Discussion

Protective immune responses to *S.* Typhi infection are complex and involve both humoral and cellular immune responses.^3^,^15^ Antibodies play a role in host defense against extracellular *Salmonella,* and bactericidal activity of serum increases with age in typhoid-endemic areas.^16^ However, because *S.* Typhi can persist intracellularly, cell-mediated immunity is critical to clearance of infection.^3^,^15^ Animal studies of *S.* Typhimurium suggest that IFN-γ CD4 and CD8 responses are important mediators in eliminating infection,^17^,^18^ and oral attenuated *Salmonella* vaccines (e.g., Ty21a and CVD 908) that are protective have been shown to stimulate strong cellular immune responses, including proliferative responses and secretion of Th1-type cytokines (e.g., IFN-γ and TNF-α) after stimulation with specific *S.* Typhi antigens.^5^,^6^ However, there are few data on cell-mediated immune responses in humans during wild-type *S.* Typhi infection, especially to purified antigens. We have previously shown that IFN-γ responses against *S.* Typhi antigens are elevated in both acute and convalescent stages of human infection compared with healthy controls,^7^ and in this report we confirm such findings using additional antigens previously identified by humoral immunoproteomic screening. We also show a CD4+ IL-17 response to infection, and the production of several other cytokines. The increased IFN-γ response seen in this analysis is consistent with other human studies.^19^ A transcriptional analysis of the peripheral blood of patients with acute typhoid fever also showed increased expression of genes associated with a response to IFN-γ compared with healthy controls.^20^ Using an ELISPOT assay, we found that the majority of the target antigens, including FliC and fimbria-associated proteins, induced a significant increase in IFN-γ responses in both acute and convalescent phases of infection compared with healthy controls. FliC is a bacterial flagellin known to induce an innate response through its activity on TLR5 or NLRC4, and its use as a diagnostic marker of acute typhoid fever has been suggested.^21^ When administered as a vaccine antigen in mice, FliC enhanced subsequent induction of IFN-γ CD4 responses and bacterial clearance in mice subsequently infected with *S.* Typhimurium.^22^ A number of fimbrial subunits and fimbria-associated proteins also induced an IFN-γ response, including StaB, SteEF, StcA, and FimF. The *staB*, *steEF*, and *stcA* are part of putative chaperone-usher-dependent fimbrial operons: *staA-G* is encoded within *Salmonella* pathogenicity island (SPI) 6 of *S.* Typhi and is rarely found in other *Salmonella* spp.^23^; *steA-G* is found in a number of *Salmonella* serotypes, but is not found in *S.* Typhimuruim^23^; *stcABCD* is found in both *S.* Typhi and *S.* Typhimurium and is thought to contribute to long-term intestinal carriage in a murine model.^24^ The *fimF* encodes the adaptor component of a mannose-sensitive type I fimbria encoded by the *fim* gene cluster, which is well conserved among *Salmonella* serotypes and mediates attachment to epithelial cells.^24^,^25^ Mutations in the *fimF* gene of *S.* Typhimurium result in a nonfimbriate phenotype.^26^

The IFN-γ responses were also seen to a putative hydrolase encoded by STY1522, phage shock protein E (*pspE*), and ABC transporter, LsrA. The *lsrA* (STY3796) is the first gene of the *lsr* (*luxS* regulated) operon, which encodes an ABC transporter for the quorum sensing molecule, autoinducer-2.^27^ It has been suggested that quorum sensing may play a role in the ability of *S.* Typhimurium to invade epithelial cells through regulation of SPI-1 and flagellar genes.^28^

In *S.* Typhi-infected humans, our group has previously shown that the majority of lymphocytes involved in IFN-γ responses to *S.* Typhi proteins, including MP, PagC, and StaF, are CD4+ cells. In this study, we extended these observations to SteE, a fimbrial subunit protein, and CsgD, a major transcriptional regulator of biofilm formation in the *S.* Typhimurium model^29^; we also show that ∼80% of IFN-γ secretion originates from CD4 cells. As the most prominent IFN-γ responses were observed with MP stimulation, we proceeded to use MP to stimulate cells for subsequent analysis of cytokine responses.

Lymphocytes of *S.* Typhi-infected patients in convalescence stimulated with MP for 48 hours generated significantly higher IFN-γ, MIP-1β, TNF-β, IL-13, and IL-9 responses than lymphocytes of the same patients in the acute phase of infection. Some of the highest fold changes were seen predominantly in IFN-γ, MIP-1β, and sCD40L, suggesting that in patients with typhoid fever, antigen-specific responses are predominantly Th1 associated. Whether these fold changes reflect an increase in cytokine expression per activated cell, or an increase in a population of cells is uncertain because the assay was based on total PBMCs. A high fold change was also seen with macrophage inflammatory protein 1-β (MIP1-β), a protein associated with recruitment of T cells and macrophages. In an *in vitro* and *in vivo* model of *Listeria monocytogenes*, MIP-1β (along with MIP-1α, RANTES, and ATAC, the latter not measured in our analysis) are co-secreted with IFN-γ and acts synergistically to drive a Th1 response.^30^ Furthermore, sCD40L stimulation has been shown to play a role in regulating Th1 responses through increased T cell stimulation and production of IFN-γ.^31^ In addition to elevations seen in Th1-associated cytokines, elevations of the Th2-associated cytokines, IL-13 and IL-9, were also present suggesting that these cytokines may act as modulators of the Th1 response. Interestingly, a number of cytokines associated with neutrophil activity were elevated above the level of detection in our assay (Supplemental Table 1), including GRO and IL-8, at both the acute and convalescent phases of illness. This observation may support previous results that found that the early host transcriptional response to *S.* Typhi infection was dominated by a neutrophil response^20^; one limitation of this analysis is that as a result of sample volume limitations, we were unable to evaluate changes in cytokine response serially over multiple incubation times. Our findings, however, add to data from previous studies of typhoid patients, which showed expression of genes associated with IFN-γ and Th1-type responses.^20^

Using intracellular staining by flow cytometry, we also showed that lymphocytes from convalescing typhoid patients stimulated for 6 hours with MP contained more CD4+ cells expressing IL-17 compared with patients in the acute stage of typhoid fever. In an experimental simian immunodeficiency virus (SIV) infection model, an impairment of the IL-17 axis results in dissemination of *S.* Typhimurium.^32^ Our findings also suggest that IL-17 may play a role during systemic *Salmonella* infection in humans, and add to prior findings in mice where the IL-17 response of ileal T lymphocytes was shown to also play an important role in the early stages of protection against *S.* Typhimurium,^33^ was induced by IL-6, and was Nod1- and Nod2-dependent.^34^ The lack of IL-17 seen in the supernatant of a 48-hour culture in our current study after stimulation may reflect IL-17 expression as an early cytokine, and that other cytokines such as IFN-γ may be induced later.^33^ The absence of changes in intracellular expression of other cytokines after 6 hours of stimulation, in contrast with their elevations as soluble forms at 48 hours, may reflect cascade induction. These observations are limited by the fact that, because of sample volume limitations, we were unable to evaluate intracellular cytokines serially at multiple incubation times.

Our study has a number of limitations, including the absence of febrile control patients with documented illness other than *S.* Typhi, and our inability to perform all analyses with each antigen in each patient because of a limited quantity of blood and antigens. Our study is also one of characterization, and because of its use of freshly harvested human blood does not include a mechanistic analysis. However, despite these limitations, our report represents one of the first descriptions of cellular and cytokine responses in humans with wild-type typhoid fever in the field. We show that in patients with typhoid fever, prominent IFN-γ responses are seen in both acute and convalescent phases of infection, the majority of which involve CD4 cells. We also show a broader Th1 response and a Th17 response in convalescence, and that these are accompanied by mild elevations in Th2 cytokines that may be modulatory. Our results also suggest that antigens used in our analysis may have some use as vaccine targets because they are recognized by cellular responses in patients recovering from typhoid fever. Similarly, these antigens may have some use as diagnostic assay targets because they induce detectable IFN-γ responses in patients with typhoid fever but not in control patients in this typhoid-endemic area.

## Supplementary Material

Supplemental Table.

## Figures and Tables

**Table 1:** Clinical features of study participants with bacteremia-confirmed typhoid fever in this study

Features	Value/characteristics
Sample size (*N*)	10
Median age in years (25th and 75th percentile)	3.3 (2.5–4.5)
Gender (male)	5
Median maximum temp (25th and 75th percentile)	39.5°C (39.1–39.8°C)
Duration of fever in days (median) (25th and 75th percentile)	5 (4–5)
Abdominal pain (*N*)	1
Constipation (*N*)	0
Coated tongue (*N*)	0
Diarrhea (*N*)	3
Vomiting (*N*)	0

**Table 2:** *Salmonella* Typhi proteins used in this study

STY no.	Annotated name	Protein
	Flagellar protein	FliC
STY0206	Chaperone protein EcpD	StaB
STY0595	Fimbria like protein FimF precursor	FimF
STY0909	Uncharacterized protein	
STY1375	Phage shock protein E	PspE
STY1522	Putative secreted hydrolase	
STY2381	Putative fimbrial subunit protein	StcA
STY3089	Fimbrial subunit	SteE
STY3090	Fimbrial subunit	SteF
STY3796	Autoinducer 2 import ATP-binding protein	LsrA
STY1179	Putative regulatory protein	CsgD
Membrane preparation	Crude membrane preparation containing at least 934 *S.* Typhi proteins	MP

**Table 3:** Cytokines responses (pg/mL) in acute and convalescent (mean ± SE of mean) samples of typhoid fever patients

	Mean acute	Mean convalescence	Relative change	*P* value
IFNγ	572.8 (393)	5482.0 (2941)	9.6	0.0492
MIP-1β	80.0 (54)	2202.6 (557)	27.5	0.0417
IL-13	17.5 (8)	198.8 (97)	11.3	0.0104
sCD40L	6.3 (6)	100.0 (32)	16	0.0354
Fractalkin	4.8 (5)	59.7 (30)	12.5	0.0101
IL-9	1.1 (1)	31.0 (10)	29.6	0.0065
TNF-β	0.82 (0.8)	27.9 (14)	34.2	0.0366
Eotaxin	0.71 (0.7)	5.5 (2)	7.8	0.0154
IL-15	0.42 (0.4)	4.0 (0.8)	9.5	0.0130
