# Supplementary material for: Cellular and Cytokine Responses to Salmonella enterica Serotype Typhi Proteins in Patients with Typhoid Fever in Bangladesh
Source: Am J Trop Med Hyg. 2014 Jun 4;90(6):1024–30. doi: 10.4269/ajtmh.13-0261 (PMC4047724; doi:10.4269/ajtmh.13-0261)
Supplement: Supplementary file 1 [file SD1.pdf]

Supplemental Table 1

Cytokine responses in acute and convalescent samples of typhoid fever patients

	Mean acute	SEM	Mean convalescent	SEM	Relative change	*P* value
EGF	17.25	11.32	23.11	23.11	1.34	0.4067
Eotaxin	0.71	0.71	5.52	1.86	7.77	0.0154
FGF-2	0.00	0.00	4.15	3.36	4.15	0.2716
Flt-3 Ligand	4.81	4.40	6.32	3.50	1.32	0.2907
Fractalkin	4.79	4.79	59.70	30.44	12.45	0.0101
G-CSF	1226.19	578.58	2738.51	2074.23	2.23	0.4311
GM-CSF	494.68	180.66	819.55	182.97	1.66	0.2235
GRO	0.00	0.00	OOR >			
IFNα2	3.49	3.49	2.33	1.36	0.67	0.4964
IFNγ	572.82	392.51	5482.00	2941.18	9.57	0.0492
IL1A	265.99	124.33	395.55	84.18	1.49	0.1957
IL1B	263.05	73.69	788.21	164.85	3.00	0.0851
IL1Ra	0.00	0.00	21.67	21.00	21.67	0.1913
IL-2	3.26	1.71	17.28	8.65	5.30	0.0935
IL-3	ND		ND			
IL-4	6.28	6.28	9.72	3.97	1.55	0.2513
IL-5	2.79	2.10	58.32	41.57	20.88	0.0562
IL-6	1884.40	1212.60	8079.68	3013.30	4.29	0.1613
IL-7	16.36	8.40	56.25	17.43	3.44	0.1571
IL-8	OOR >		OOR >			
IL-9	1.06	1.06	31.34	10.22	29.56	0.0065
IL-10	53.53	38.80	150.40	29.30	2.81	0.1206
IL12 (p40)	47.04	35.88	89.02	16.76	1.89	0.1277
IL12 (p70)	3.52	1.44	8.50	1.67	2.41	0.1215
IL-13	17.54	8.37	198.76	96.51	11.33	0.0104
IL-15	0.42	0.42	3.95	0.75	9.48	0.0130
IL-17	4.81	4.81	5.71	3.12	1.19	0.2051
IP 10	112.81	53.99	516.92	155.47	4.58	0.1057
MCP-1	0.00	0.00	0.00	0.00		
MCP-3	0.00	0.00	279.68	279.68	279.68	0.2113
MDC	0.00	0.00	0.00	0.00		
MIP-1α	OOR >		OOR >			
MIP-1β	79.66	53.68	2202.62	556.99	27.65	0.0417
sCD40L	6.26	6.26	99.96	31.87	15.96	0.0354
sIL-2Ra	5.03	3.28	67.88	25.71	13.49	0.0561
TGFα	0.00	0.00	3.80	2.20	3.80	0.1005
TNFα	910.43	500.57	978.84	210.93	1.08	0.2625
TNFβ	0.82	0.82	27.90	14.31	34.16	0.0366
VEGF	9.10	9.10	48.53	19.94	5.33	0.0718

ND = not detected; OOR > = out of range high.
